# The genome of the solitary bee *Tetrapedia diversipes* (Hymenoptera, Apidae)

**DOI:** 10.1093/g3journal/jkae264

**Published:** 2024-12-24

**Authors:** Priscila K F Santos, Natalia de Souza Araujo, Elaine Françoso, John H Werren, Karen M Kapheim, Maria Cristina Arias

**Affiliations:** Departamento de Genética e Biologia Evolutiva, Instituto de Biociências, Universidade de São Paulo, Rua do Matão, 277, CEP 05508-090, São Paulo, SP, Brazil; Department of Biology, Utah State University, 5305 Old Main Hill, Logan, UT 84322, USA; Department of Evolutionary Biology and Ecology, Université Libre de Bruxelles, Av. Franklin Roosevelt 50, 1050 Bruxelles, Belgium; Department of Biological Sciences, School of Life Sciences and the Environment, Royal Holloway University of London, Egham Hill, Egham TW20 0EX, UK; Department of Biology, University of Rochester, 210 Hutchison Rd, Rochester, NY 14620, USA; Department of Biology, Utah State University, 5305 Old Main Hill, Logan, UT 84322, USA; Departamento de Genética e Biologia Evolutiva, Instituto de Biociências, Universidade de São Paulo, Rua do Matão, 277, CEP 05508-090, São Paulo, SP, Brazil

**Keywords:** Neotropical, genome sequencing, de novo assembly, oil-collecting bee, *Tetrapedia diversipes*

## Abstract

*Tetrapedia diversipes* is a Neotropical solitary bee commonly found in trap-nests, known for its morphological adaptations for floral oil collection and prepupal diapause during the cold and dry season. Here, we present the genome assembly of *T. diversipes* (332 Mbp), comprising 2,575 scaffolds, with 15,028 predicted protein-coding genes. Repetitive elements constitute 38.68% of the genome, notably Class II transposable elements. An investigation into lateral gene transfers identified a low frequency (0.037%) of nuclear copies of mitochondrial DNA and 18 candidate regions from bacterial origins. Furthermore, the annotation of 3 scaffolds reveals the presence of the *Wolbachia* endosymbiont genome, confirming the infection by 2 strains in *T. diversipes* populations. This genome contributes valuable insights into Neotropical bee genomics, offering a resource for comparative studies and enhancing our understanding of the molecular basis of solitary bee adaptations and interactions.

## Introduction

*Tetrapedia diversipes* is a solitary bee from the Neotropical region. Females construct their nests in preexisting cavities, such as abandoned beetle holes (Alves-dos-Santos *et al*. 2002). Due to the female's philopatric behavior, the reuse of nests by subsequent generations is common, resulting in a high abundance of this species in trap-nests (Cordeiro *et al*. 2019; Santos *et al*. 2020). Consequently, it serves as an excellent biological model due to its facilitated sampling and maintenance.

**Fig. 1.**
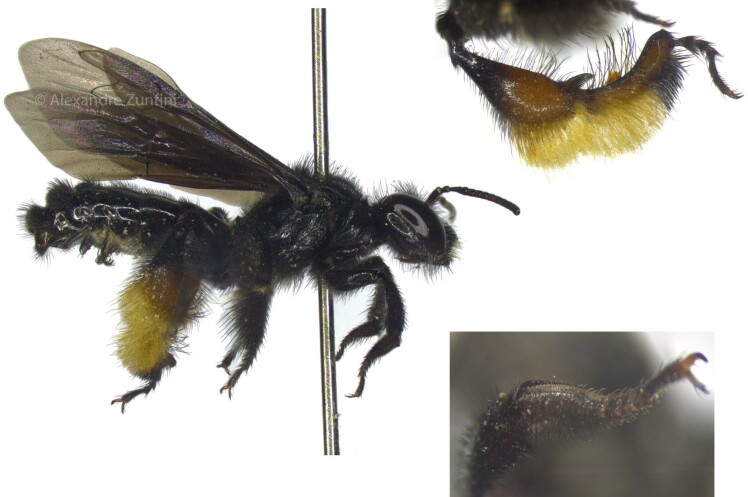
Male specimen of *T. diversipes* collected from trap-nests maintained at the Bee Laboratory (USP, São Paulo, Brazil). Details of the posterior and anterior legs are highlighted at the top and bottom right, respectively. Both legs contain adaptations for oil collection (Alves-dos-Santos *et al*. 2002). Photograph by Alexandre Zuntini, used with permission.

Both males and females of this species have morphological adaptations for collecting floral oils (Alves-dos-Santos *et al*. 2002) (Fig. 1). While males likely utilize these oils for mating purposes (Cappellari *et al.* 2012), females employ them in nest construction by mixing with soil and for provisioning larvae by mixing with pollen (Alves-dos-Santos *et al*. 2002). In addition to its unique adaptations for floral oil collection, *T. diversipes* exhibits other remarkable biological traits. This includes entering diapause during the colder and drier months in the subtropical region of Brazil, sharing a common ancestor with the sub-social lineages from Xylocopinae, and engaging in a host–parasite relationship with the cleptoparasitic bee *Coelioxoides waltheriae* (Alves-dos-Santos *et al*. 2002; Bossert *et al*. 2018; Santos *et al*. 2018).

Despite advances in sequencing technologies and the growing number of genomes from nonmodel organisms in databases, bee species from the Neotropical region remain significantly underrepresented. Although 60% of social bee species are endemic to the Neotropics (Noll *et al*. 2018), only 8 social bee species (*Bombus dahlbomii*, *Euglossa dilemma*, *Frieseomelitta varia*, *Megalopta genalis*, *Melipona beecheii*, *Melipona bicolor*, *Melipona quadrifasciata*, and *Tetragonisca angustula*) and 1 solitary species (*Eufriesea mexicana*) from this region are included among the 112 bee genomes available on GenBank (accessed on 2024 September 20).

Here, we present the genome of *T. diversipes* and its key characteristics. The genome spans 332 Mbp, distributed across 2,575 scaffolds, with 38.68% consisting of repetitive elements. Evidence suggesting possible lateral gene transfer (LGT) events from mitochondrial DNA and endosymbionts was also detected. A total of 15,028 protein-coding genes were predicted through annotation. This genome will be a valuable resource for future comparative and evolutionary studies in bee research.

## Materials and methods

### Sample collection and sequencing

*Tetrapedia diversipes* larvae and adults were collected from trap-nests maintained at the Bee Laboratory [University of São Paulo (USP), São Paulo, Brazil]. For male selection among the larvae, 10 individuals were screened through 16 microsatellite loci following standardized conditions (Arias *et al*. 2016), being male hemizygotes for all loci. Additionally, the mitochondrial gene cytochrome c oxidase I was sequenced to differentiate *T. diversipes* larvae from its cleptoparasitic bee, *C. waltheriae*. For the Illumina sequencing, DNA was isolated from whole larva body following a phenol–chloroform protocol (Bonasio *et al*. 2010; Standage *et al*. 2016). DNA quantification was performed using a Qubit 2.0 fluorometer system (Invitrogen), and DNA quality was verified by agarose gel electrophoresis. The samples with the highest DNA integrity and quantity were sent to Macrogen (South Korea) for paired-end library construction and sequencing. For 100-bp short-read sequencing, 3 different libraries were constructed. One male larva was used for the TruSeq DNA PCR-Free (350 bp insert) library, and 2 male larvae were used for each Nextera Mate Pair (Gel-Plus) library of 3 and 8 kb insert sizes. All libraries were sequenced using 1 lane of the Illumina HiSeq2500 platform. For long-read sequencing, DNA was obtained from the entire body, excluding the abdomen, of 2 newborn adult males. The same DNA extraction protocol was used. One 20-kb SMRTbell Templates library was constructed and sequenced in 3 SMRT cells of the Pacbio Sequel system. A summary is presented in Supplementary Table 1.

### Data preprocessing and genome assembly

The FASTQC-v0.11.6 software (Andrews 2010) was utilized to check the quality of Illumina sequencing. Illumina reads smaller than 36 bp and with a Phred score below 20 (for shotgun library) or 15 (for mate-pair libraries) were removed using Trimmomatic-0.36 (Bolger *et al*. 2014). PacBio subreads are output free of adapters and were corrected through overlapping using Canu-v1.8 software (Koren *et al*. 2017). Short and long reads were combined in a hybrid de novo genome assembly using MaSuRCA-v3.2.6 (Zimin *et al*. 2013) with default parameters. Genomic statistics (total number of scaffolds, contigs, largest scaffold, N50, total length, and N's per 100 kb) and quality were assessed using Quast-v5.2.0 (Gurevich *et al*. 2013), and completeness was evaluated using the Benchmarking Universal Single-Copy Orthologs (BUSCO)-v5.3.2 software (Simao *et al*. 2015). For this, the Hymenoptera database of single-copy orthologs containing 5,991 genes (September 2022) was used.

### Prediction and annotation of protein-coding genes

Genome annotation was performed using Maker-v2.31.10 (Holt and Yandell 2011) following the tutorial at http://weatherby.genetics.utah.edu/MAKER/wiki/index.php/MAKER_Tutorial_for_WGS_Assembly_and_Annotation_Winter_School_2018 and https://gist.github.com/darencard/bb1001ac1532dd4225b030cf0cd61ce2 (accessed in September 2018). Initially, RepeatMasker-v4.0 (Smit *et al*. 2015) was used within Maker to mask low complexity and repetitive regions of the genome based on Repbase (Jurka *et al*. 2005) repeat library from all available species (April 2019). Gene prediction proceeded in 2 rounds. In the first round, “est2genome” and “protein2genome” options were enabled (set to 1), using a combination of the de novo assembled transcriptome of *T. diversipes* (Santos *et al*. 2018) and bee protein sequences from the UniRef90 database (April 2019) (Suzek *et al*. 2007) as input. To build the bee proteins database, we accessed the UniProt website, selected the UniRef database, searched for Apoidea, and downloaded the fasta sequences from the 90% cluster.

In the second round, SNAP (Korf 2004) and Augustus (Stanke and Waack 2003) were trained to refine gene prediction. Gene models predicted in the first round with an annotation edit distance < 0.25 and protein length of 50 or more amino acids served as input to train SNAP. Augustus was trained inside BUSCO-v3.0.2 using the Hymenoptera database (September 2018 containing 4,415 genes) with the “–long” argument to optimize the hidden Markov model search model. After training, Augustus and SNAP were used to predict the gene models, with the “est2genome” and “protein2genome” options set to 0. This training and predicting cycle was repeated 3 times until the best results were observed, indicated by larger average gene size and more predicted proteins annotated with BUSCO. Finally, the functional annotation of *T. diversipes*’ final gene set was generated by running Blastp (Johnson *et al*. 2008) using the SwissProt (April 2019) (Bairoch and Apweiler 1996) and InterproScan-v5.34-73.0 (Hunter *et al*. 2009) databases.

### Repetitive elements

To identify transposable elements (TEs) in the genome, we developed a comprehensive custom library for *T. diversipes* using multiple strategies. Initially, 3 independent repeat libraries were created using RepeatModeler-v.open-1.0.11, TransposonPSI-v2.2.26, and LTRharvest (included in GenomeTools-v1.5.8—Gremme *et al*. 2013). Libraries from TransposonPTS and LTRdigest were then classified with RepeatClassifier (included in RepeatModeler). All classified libraries were concatenated and then merged based on similarity (≥80% identity) using USEARCH-v11.0.667 (Edgar 2010) to create a nonredundant library, which was classified once again with RepeatClassifier. Finally, the custom library was merged with the *Apis mellifera* repeat library contained in Dfam-v3.1, available within the RepeatMasker-v4.1.0 software (Smit *et al*. 2015). A genome search for all TEs was conducted using our merged database with RepeatMasker. Statistical analysis and figures were generated in R-v3.4.3. Detailed parameters and scripts from this pipeline are available at https://github.com/nat2bee/repetitive_elements_pipeline.

### Lateral gene transfers

Nuclear copies of mitochondrial DNA (NUMTs) were identified through a BLASTN search of the complete mitochondrial genome (mtGenome) of *T. diversipes* (Françoso *et al*. 2020; GenBank accession number: MN732885.1), which was used as the reference sequence against the nuclear genome described here. The search was conducted using Geneious-v2023.0.4 software (https://www.geneious.com) with default parameters and a low complexity filter. The methodology was developed based on Behura (2007) and Nacer and Raposo do Amaral (2017). NUMTs candidates were manually inspected. Only hits with an expect value equal to or smaller than 10^−5^ and length sequences over 300 bp with pairwise identity between 75% and 98% were considered. Hits with similarities over 98% were discarded as false positives.

To identify LGTs from bacterial origins, a preliminary screen for bacterial scaffolds was performed following Wheeler *et al*. (2013). Subsequently, each scaffold was divided into 1 kb fragments, and each fragment was used as query in a BLASTN search against a custom database containing 2,100 different bacterial species (Supplementary Table 2). LGT candidates at the beginning position of a large scaffold were considered as misassembled. A second BLASTN search was carried out against the *T. diversipes* genome free of contaminants available at GenBank (accession number: GCA_033822845.1) and the nonredundant Procaryotae GenBank database to confirm the LGTs. The final LGTs were considered sequences present in the *T. diversipes* genome with bitscore = 0 against the animal database and bitscore > 75 against the bacterial database.

### *Wolbachia* endosymbiont of *T. diversipes*

We utilized RAST Server-v2.0 (Aziz *et al*. 2008) to annotate the genes of 3 scaffolds identified as originating from *Wolbachia* endosymbiont. Subsequently, we compared the 5 genes (*gatB*, *coxA*, *hcpA*, *fbpA*, and *ftsZ*) part of the multilocus sequence typing (MLST; Baldo *et al*. 2006) with the PubMLST database (Jolley *et al*. 2018) to infer the *Wolbachia* alleles.

### Large language models usage

ChatGPT-v3.5 was used to correct grammar and improve syntax throughout the text. The following prompt was given: “Correct the grammar [text pasted].”

## Results and discussion

### Genomic assembly and annotation

A total of 509,778,166 reads and 1,190,034 long reads from Illumina and PacBio sequencing, respectively, were employed for the assembly of the *T. diversipes* genome. The assembled genome, spanning 332,342,503 bp (332 Mbp), was distributed among 2,575 contigs (NCBI accession GCA_033822845.1). The largest scaffold measured 2,952,783 bp, the N50 for contigs > 200 bp was 395,597 bp, and the quantity of N's per 100 kb was 497.59 bp. Regarding the 5,991 single-copy hymenoptera orthologues, 93.9% were complete and single copy, 1.1% were complete and duplicated, 1.0% were fragmented, and 4.0% were missing in the *T. diversipes* genome assembly.

**Fig. 2.**
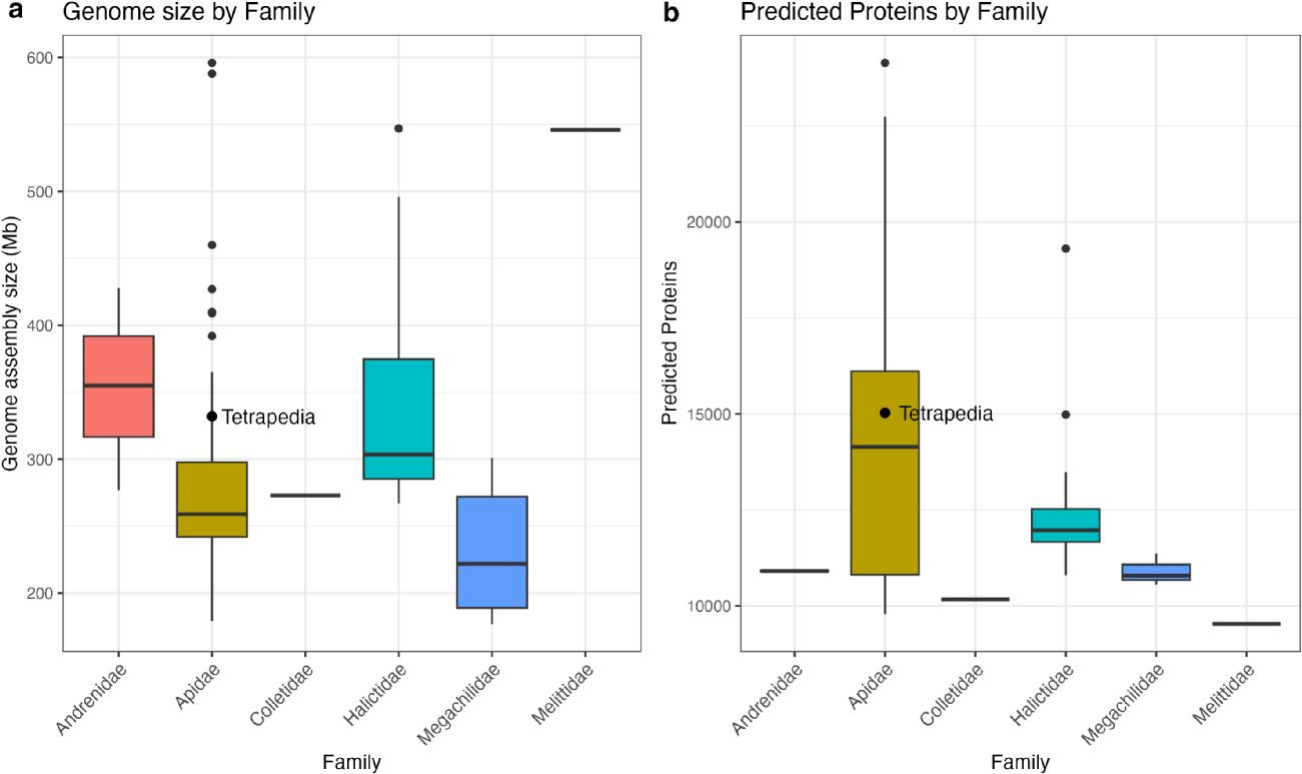
Boxplots showing a) genome assembly sizes and b) number of predicted proteins across bee families. *Tetrapedia diversipes* is highlighted within the Apidae family. Families with only a median line in the boxplot indicate that only 1 species is representing that family.

Gene prediction identified 15,028 protein-coding sequences with an average size of 493.6 bp (SE ± 4.4 bp). Among these sequences, 10,437 had matches against the SwissProt database. The final gene set fasta file and the gff annotation file can be accessed at https://github.com/pkfsantos/Tetrapedia_diversipes_genome. In comparison with the Hymenoptera database in BUSCO, the entire gene set contained 89.9% of completed single copy, 1.1% of completed and duplicated, 3.1% of fragmented, and 5.9% of missing genes. The genome size, number of predicted genes, and genome quantitative and qualitative metrics fall within the expected range compared with other bee species (Fig. 2; Supplementary Table 3).

### Repetitive elements

**Fig. 3.**
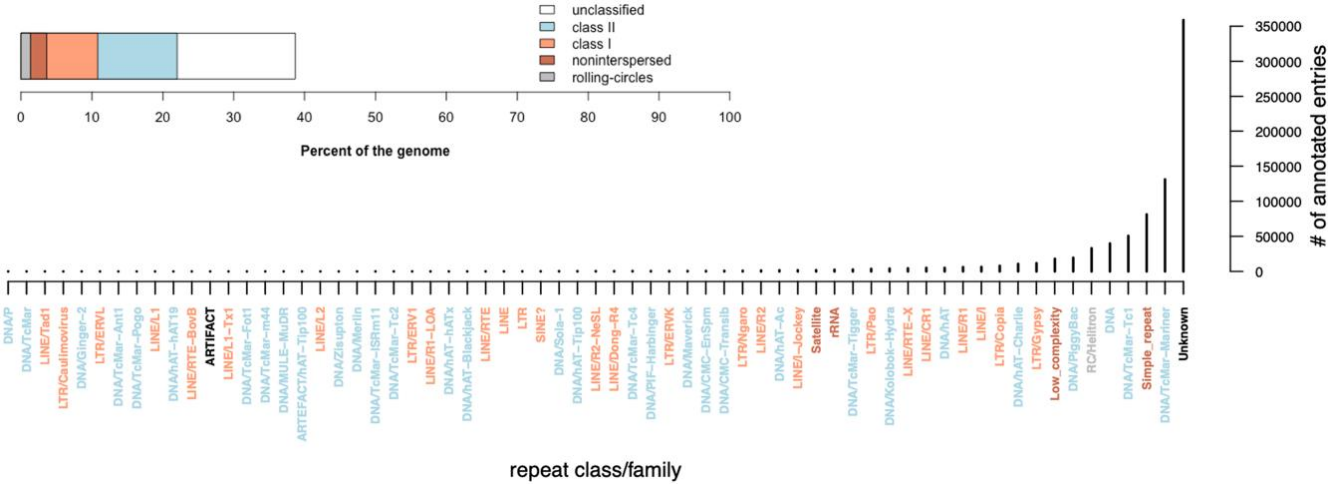
Repetitive elements identified in the *T. diversipes* genome. Top: The proportion of all repetitive elements in the genome is split by class and type. Bottom: Distribution of repeats and TE families number identified. Colors correspond to the element's major class/type. Sequences labeled as “artifact” may resemble TEs but are not considered real, functional elements, while those labeled as “unclassified” are recognized as TEs but cannot be reliably assigned to any known TE family or superfamily.

The amount of repetitive elements reported in bee genomes is an intriguing feature that seems to be related to the level of social organization; the more complex the social organization level (e.g. highly eusocial), the smaller the proportion of repetitive DNA (<20% or less) in the genome (Kapheim *et al*. 2015). Specifically, TEs might have multiple regulatory functions, including epigenetic regulation, gene expression, splicing, and genomic rearrangements (Kazazian 2004; Slotkin and Martienssen 2007; Bourque *et al*. 2018). In *T. diversipes*, the repetitive elements accounted for 38.68% of the genome, including 2.32% of other noninterspersed repeats (Fig. 3). Class II TEs (elements capable of moving from one site to another in the genome in a *cut-and-paste* transposition mechanism—DNA transposon) were the most frequent in the genome, accounting for 12.54% of the repeats. Among them, the most frequent superfamilies were the Tc1/IS630/Pogo (7.83%), Helitron (1.31%), PiggyBac (0.81%), and hobo-Activator (0.76%). Class I elements (elements that are transcribed into RNA as an intermediate before their reversed transcription into DNA and incorporation in a copy-and-paste transposition mechanism—retrotransposon) summed up to 7.17% of the genome, with short interspersed nuclear elements (SINEs) and long interspersed nuclear elements (LINEs) accounting for 0.01 and 3.45% of the repeats, respectively. Long terminal repeats (LTRs) elements were the most abundant TE, comprising 3.71% of the genome. In total, TEs were classified into 59 known classes/families categories (Fig. 3). Compared with 10 bee genomes assessed by Kapheim *et al*. (2015), the repetitive elements content in the *T. diversipes* genome is high (38.68%). Only 2 other solitary bees, *E. mexicana* (49%) and *Megachile rotundata* (43.23%), have a higher quantity of repetitive elements (Supplementary Table 4.1; Kapheim *et al*. 2015).

A portion of these repetitive elements (4%) occurs around gene regions and could affect gene expression dynamics by interacting with the promoter regions and transcription factors (Supplementary Table 4; Supplementary Fig. 1;  Bourque *et al*. 2018; Uzunović *et al.* 2019). Regions within 5-kb window upstream or downstream of a gene location were enriched for elements from the LINE, LTR, Maverick, and terminal inverted repeats (TIRs) groups (Supplementary Table 4.2, Fisher's exact test *P* < 0.01). On the other hand, 12 TE categories were not found within these regions (ARTEFACT/hAT-Tip100, DNA/Ginger-2, DNA/hAT-hAT19, DNA/MULE-MuDR, DNA/P, DNA/TcMar, DNA/TcMar-Ant1, DNA/TcMar-Pogo, LINE/RTE-BovB, LINE/Tad1, LTR/caulimovirus, and LTR/ERVL).

### LGT—NUMTs and bacteria–host origin

LGT refers to the transfer of DNA between organisms without involving sexual reproduction. In animals, the most common transfers occur between mitochondrial and nuclear DNA (NUMTs), as well as between intracellular bacteria and their host (Klasson *et al*. 2014). We investigated both types of LGTs in the *T. diversipes* genome.

NUMTs are spread among eukaryotes, comprising around 0.1% of the nuclear genome. Usually, the larger the genome, the greater the frequency of NUMTs (Hazkani-Covo *et al*. 2010). A total of 182 possible NUMT sequences were found based on the mtGenome of *T. diversipes*, representing 0.037% of the nuclear genome. This amount of NUMTs found is lower than the 0.08% described for *A. mellifera* (Behura 2007), despite the *T. diversipes* genome being larger. The length of NUMT sequences ranged from 300 to 2,077 bp, and the pairwise identity ranged from 75.1 to 97.3% (Supplementary Table 5). NUMTs appear to have originated equally from all mtGenome regions (Supplementary Table 5; Supplementary Fig. 2), with the exception of the AT-rich region and adjacent, which presented fewer NUMTs when compared with the other regions. Given that NUMTs are predominantly comprised of neutral regions, the notable variability observed in pairwise identity for each gene suggests the likelihood of multiple nuclear insertions.

After excluding 26 scaffolds identified as entirely of bacterial origin, the genome scan for bacterial LGTs returned 36 candidate regions across 26 different scaffolds. Out of the 36 candidates, 15 were trimmed or removed through the contamination exclusion pipeline performed by NCBI, 1 had bitscore > 0 against the animal database, and 2 are unlikely LGTs because the region that matches to bacterial material is located at the end of the scaffold and might be the result of misassembly. The remaining 18 regions varied from 45 to 217 bp, with 9 fragments identified as being from *Wolbachia* spp., the most common donor of LGTs (Supplementary Table 6).

Gene transfer from *Wolbachia*, while commonly observed among arthropods, tends to be evolutionary recent and species-specific (Hotopp *et al*. 2007). Approximately one-third of invertebrate genomes exhibit recent *Wolbachia* gene insertions (Werren *et al*. 2008). The prevalence of *Wolbachia* extends across various bee species (Gerth *et al*. 2013, 2015). Moreover, LGTs from this endosymbiont to host genomes have been identified not only in bees but also in other Hymenoptera, such as ants (Dhaygude *et al*. 2019) and wasps (Werren *et al*. 2010).

### *Wolbachia* endosymbiont of *T. diversipes*

In addition to the LGTs from *Wolbachia* in the nuclear genome, 3 scaffolds were identified as being from the endosymbiont genome. The 3 scaffolds from *Wolbachia* are 1,851,538, 705,933, and 25,845 bp in size and can be found at: https://github.com/pkfsantos/Tetrapedia_diversipes_genome. Santos *et al*. (2022) screened for *Wolbachia* in 5 populations of *T. diversipes* and found that most individuals were infected by 2 different strains concurrently, possibly from 2 different supergroups A and B. The MLST genes, expected to be present in a single copy in the endosymbiont genomes, are commonly used to identify *Wolbachia* strains (Baldo *et al*. 2006). In our analysis, we identified 2 copies of each MLST gene in 2 *Wolbachia* scaffolds (Supplementary Table 7). Each gene copy corresponds to different alleles when compared against the PubMLST database (Supplementary Table 8). These data corroborate the previous findings of 2 strains infecting *T. diversipes* individuals in a population assay. However, all gene copies but one (from *ftsZ* gene) were found in the longest scaffold, suggesting a misassembly that combined the genomes of the 2 strains.

## Conclusions

The genome of the solitary bee *T. diversipes* comprises 15,028 predicted protein-coding genes distributed across 332 Mbp. Similar to other solitary bee species, it is rich in repetitive elements. Additionally, our findings suggest the possible occurrence of LGT events involving both the mitochondrial and endosymbiont genomes. The data confirm previous findings of *T. diversipes* population infection by 2 *Wolbachia* strains.

## Supplementary Material

jkae264_Supplementary_Data

## Data Availability

The raw data for the *T. diversipes* genome assembly are available under the BioProject PRJNA866601 in the NCBI database. The assembly can be accessed on GenBank—NCBI with the accession number JAOPTO000000000—GCA_033822845.1. The mtGenome can be located under MN732885.1. Steps and commands used in the genome assembly and annotation process, R code for Fig. 2, the 3 *Wolbachia* scaffolds, along with the *T. diversipes* gene set and genome annotation file, are accessible at https://github.com/pkfsantos/Tetrapedia_diversipes_genome. The pipeline for the repetitive elements analysis can be found at https://github.com/nat2bee/repetitive_elements_pipeline. Any other analysis details will be available upon request. Supplemental material available at G3 online.
